# How video calls affect mimicry and trust during interactions

**DOI:** 10.1098/rstb.2021.0484

**Published:** 2023-04-24

**Authors:** Fabiola Diana, Oscar E. Juárez-Mora, Wouter Boekel, Ruud Hortensius, Mariska E. Kret

**Affiliations:** ^1^ Comparative Psychology and Affective Neuroscience Laboratory, Department of Cognitive Psychology, Leiden University, Wassenaarseweg 52, 2333 AK, Leiden, The Netherlands; ^2^ Leiden Institute for Brain and Cognition (LIBC), Leiden University, Wassenaarseweg 52, 2333 AK, Leiden, The Netherlands; ^3^ Laboratorio de Ecología de La Conducta, Instituto de Fisiología, Benemérita Universidad Autónoma de Puebla, Puebla, Puebla 72530, Mexico; ^4^ Department of Psychology, Utrecht University, Utrecht, Heidelberglaan 1, 3584 CS Utrecht, The Netherlands

**Keywords:** behavioural mimicry, trust, video calls, yawning, scratching

## Abstract

Many social species, humans included, mimic emotional expressions, with important consequences for social bonding. Although humans increasingly interact via video calls, little is known about the effect of these online interactions on the mimicry of scratching and yawning, and their linkage with trust. The current study investigated whether mimicry and trust are affected by these new communication media. Using participant-confederate dyads (*n* = 27), we tested the mimicry of four behaviours across three different conditions: watching a pre-recorded video, online video call, and face-to-face. We measured mimicry of target behaviours frequently observed in emotional situations, yawn and scratch and control behaviours, lip-bite and face-touch. In addition, trust in the confederate was assessed via a trust game. Our study revealed that (i) mimicry and trust did not differ between face-to-face and video calls, but were significantly lower in the pre-recorded condition; and (ii) target behaviours were significantly more mimicked than the control behaviours. This negative relationship can possibly be explained by the negative connotation usually associated with the behaviours included in this study. Overall, this study showed that video calls might provide enough interaction cues for mimicry to occur in our student population and during interactions between strangers.

This article is part of a discussion meeting issue ‘Face2face: advancing the science of social interaction’.

## 1. Introduction

Video calls were already a widespread means of communication in various work fields, but their usage in maintaining personal relationships has had an unprecedented spike during the COVID-19 pandemic. Human social interactions are intriguingly complex. They require not only moment-to-moment tuning of explicit signals such as facial expressions, bodily signals, and tone of voice but also of subtle implicit cues that are autonomic (i.e. not under conscious control), such as changes in pupil size or blush [1]. This plethora of signals sent back and forth is also referred to as mimicry, the tendency to automatically mimic and synchronize movements, facial expressions, gestures and eye-gaze ([2], for an extensive review see [3]). Mimicry has been suggested to be a mechanism that drives our ability to share others' emotions with others [2,4]. Together with cognitive processes, mimicking others’ behaviour may help inform us about their intentions and feelings, potentially influencing whether we perceive someone as trustworthy and likeable or not [3,5,6]. To what extent do video calls, during which we have limited access to expressions, influence mimicry and person perception (e.g. trustworthiness)? As our dependence on video calls will most likely increase in the future, the impact of this medium on our social interactions deserves further investigation. The current study aims to investigate whether and to what extent video calls influence mimicry and its linkage with trust.

As a social species, cooperation represents a necessary element for human life (i.e. cooperative hunting, food sharing, reciprocation, alloparenting) to increase the likelihood of survival of the individual and the group [7]. In a competitive situation, the choice to cooperate may turn into a social dilemma: cooperative behaviours maximize social welfare, but defection only favours the single individual at the cost of the others [8]. Defecting for your own gain is tempting in such a dilemma, and voluntarily cooperating depends on the confidence that all parties involved will reciprocate [9]. Although motives for cooperation may be present in the first place, the fear that others will not cooperate is likely to result in the choice not to cooperate [10]. As such, trust in reciprocity seems critical for the success of a cooperative task that involves the risk of deceit [9,11,12]. Among the several paradigms employed to investigate social-decision making in a cooperative setting [13], the Trust Game stands out as the most suitable to assess trust decisions [14]: two individuals, the trustor and the trustee, are given a monetary incentive. The trustor can choose to send some part of his budget to the trustee, who can decide whether or how much money to send back. Both the trustor and the trustee can obtain a higher outcome when the investor maximally invests because investments get tripled (see Material and methods). Previous research has shown that investing in the trustee relies on the trustor's characteristics (e.g. social anxiety [15]; risk aversion [16]; empathy [17]) and the trustee's characteristics, including reputation [17] and attractiveness [18]. Even so, what makes us sure the counterpart will reciprocate? What signals do we rely on to decide whether to trust or not? Trust is often intuitive and may reflect a ‘*gut feeling*’ based on several partners' physical features or behaviours that can impact our investment decision [19].

This ‘gut feeling’ is often the result of an affect-based nonconscious evaluation. Across species, this evaluation is based on different emotional sources, such as posture [20,21], smell [22] and vocalizations [23]. The face plays a major part in non-verbal interaction as it conveys several signals and cues contributing to social perception. Previous research has shown that smiling fosters cooperative intentions [24]. Although cooperation was lower when participants were playing with angry partners [25], smiling participants were more willing to cooperate and elicit more cooperation from their partners than participants expressing contempt [24]. Although facial expressions are a salient stimulus, not all expressions reflect genuine emotions and intentions. For instance, we can fake a smile to appear trustworthy and gain benefits that would otherwise be denied [26]. Unfortunately for non-cooperators, emotions and intentions are not expressed only by the face and its muscle actions: we are exchanging numerous autonomic cues outside our conscious control (i.e. pupil size, blushing) that are slowly starting to receive the attention of the scientific field of emotion perception [1].

Some frequently observed behaviours in emotional situations appear to be particularly contagious: yawning and scratching [27,28]. Yawning is characterized by a powerful stretch of the jaw with a deep inspiration, followed by a shorter exhalation with a passive jaw closure [27]. Mammals and most other vertebrates yawn, and humans start yawning already in the prenatal phase [29]. Why we yawn is still a debated question. Numerous functions have been proposed for yawning, such as stimulating and facilitating arousal during state changes [30], increasing mental efficiency [31,32], releasing tension [33] and nonconsciously communicating psychological stress [34] or drowsiness [35,36]. While it is true that yawns occur in the transitions between rest and wakefulness [35], recent research has hypothesized that yawning could be triggered by mental and physical stress [37–39]. This proposal resonates well with human studies observing increased yawning rates before anxiety-provoking and stressful situations [29] and increased cortisol levels after yawning, although the study was tested in small sample size [33]. Acute physical stress was found to significantly modulate yawning response [40]. Beyond human studies, the relationship between stress and yawning has been partially confirmed in animal research. A study on *Theropithecus gelada* (geladas) investigated the effect of three types of yawn display [41]. They found that two displays were more linked with affiliative intents, but one display was significantly associated with agonistic and tension situations [41]. Together, these findings suggest not only that yawning may convey a message with negative connotations related to the stress of the individuals, but also that yawning intensities may have multiple communicative effects. Yawning is remarkably contagious: simply watching, reading, or thinking about it can initiate a response that, once started, cannot be completely suppressed. Contagious yawning has been observed in several mammals [42–48] and some birds [49]. Based on the functions hypothesized for spontaneous yawning, contagious yawning has been proposed as a form of nonconscious communication to coordinate arousal, synchronize behaviours and enhance vigilance within the group [29,32]. In fact, previous research reported an effect of familiarity and emotional proximity of the expressor on contagiousness ([43,45,50,51], but see [47]). Yawn contagion seems to connote an underlying connection between individuals and suggests this phenomenon might rely on motor behaviour and more subtle emotional pathways [43]. These observations drove researchers to propose contagious yawning as a primitive form of empathetic behaviour ([29,50] but see [52]).

Another important contagious behaviour is scratching, described as the conclusive action to the irritating sensation of itch [53]. Scratching shares features with yawning, the most important being the high contagiousness: images and videos of scratching, as well as hearing or seeing someone scratch, increases the sensation of itching and the urge to scratch in the observer [28]. Contagious scratching has been demonstrated in non-human animals [54–56]. Mimicking scratching may have the primary evolutionary advantage of keeping parasites away [57]: if one group member is scratching, it is beneficial for the others to scratch as well. As for yawning, it seems that contagious scratching conveys a social message [58]. It is frequently associated with the presence of psychological and physiological stress [47,59]. While a positive social bond seems to play an important role in contagious yawning, Laméris *et al.* [55] found the opposite: in a tense situation, scratch contagion in orangutans was particularly observed between weakly bonded group mates [55]. This suggests that the familiarity bias is context-dependent (e.g. tense versus relaxed environments). The relationship between scratching and nervousness has been observed in humans too [60]. In fact, it seems that humans not only tend to scratch when being tense but also feel nervous when exposed to scratching agents [61]. Even though contagious yawning and scratching share some features, it is still unknown whether the same mechanism drives them. What the aforementioned literature suggests is that both yawning and scratching have been associated with stressful and tense situations [34,61]. The morphology of yawning and scratching is indistinguishable, whether spontaneous or contagious [62]. Based on the cumulative properties of evolution, it is reasonable to expect that they also share similar functional properties and that the factors triggering the spontaneous behaviour would also affect the contagious one [62]. This claim has been confirmed by several studies [40,63,64]. As such, literature suggests that not only spontaneous but also contagious scratching and yawning may be associated with negative situations. As other non-verbal behaviours (e.g. facial expressions, blushing, pupil dilation) may affect our social decisions, a logical question is whether scratching and yawning also influence the social perception of others (e.g. trust).

The occurrence of automatic mimicry has been extensively established for several emotional and non-emotional behaviours [29]. Lakin *et al.* [65] proposed nonconscious mimicry as an affiliative social glue. This idea gained credibility since mimicry of emotional facial expressions enhances linking and affiliation [26,66]. Mimicry also resulted in a greater rate towards the ingroup compared to the outgroup, suggesting that it is more beneficial to cooperate with close individuals [1,42]. These findings have been extended to pupil dilation: the nonconscious mimicry of pupil dilation was related to a more pleasant and trustworthy perception of the interaction partner [6,19,67]. Such results would suggest that mimicry in humans may be bound to positive behaviours to increase cooperation and affiliation principally within the group [65,68]. Another line of research proposed that the effect of mimicry may vary based on the social context as well as the behaviours that are mimicked. While pupil dilation mimicry has been associated with increased trust in the partner [19], mimicry of constricted pupils has been shown to decrease trust [19,69]. Similarly, a study on yawn contagion showed that different yawning displays might be associated with affiliative *and* agonistic intent [41]. The mimicry of scratching and yawning has been noted to be greater during tense situations among individuals who are not socially close [55,70]. A recent study showed not only that mimicry was greater when outgroup faces – framed as threatening—were shown but also that outgroup mimicry was associated with activation in different brain areas compared to smile mimicry [70]. Finally, research on virtual agents failed to find an effect of mimicry on the perceived likability of the agent [71,72]. The substantially mixed results of the literature showed that a clear function of mimicry has not yet been established. Solely framing mimicry as a social glue could be reductive given the realm of possible behaviours and social context still underexplored in automatic mimicry research.

A closer look at the literature on mimicry reveals several shortcomings, and many questions regarding its impact on social perception (e.g. trust) remain to be addressed. The present study investigated whether mimicry and trust are affected by the different social contexts provided during video calls. In particular, we focused on the mimicry of scratching and yawning to explore how it changes between the different social settings of video calls and face-to-face interactions. We tested whether being in a video call or face-to-face interaction modulated the relationship between mimicry of scratching and yawning, and trust. Most research has used computer tasks to investigate emotional mimicry [11,73] and contagious yawning ([74]; electronic supplementary material, table S1). Here, we tested mimicry in an ecologically valid setting where participants played multiple rounds of a trust game with a confederate in three conditions: face-to-face, video call and pre-recorded video. The confederates were instructed to perform two target behaviours (yawning, scratching) and two control behaviours (lip-biting, face-touching), one for each trial of the experiment. All laboratory sessions were recorded to investigate mimicry, and two independent raters coded all behaviours after the experiment. To our knowledge, no prior studies have investigated the mimicry of different behaviours in the context of a video call. Unlike face-to-face interaction, non-verbal behaviours (e.g. facial movement, posture, eye contact) can be less effective or ambiguous in an online call [75,76]. Therefore, our first hypothesis was that the more direct the communication is, the more mimicry will occur. We predicted mimicry to decrease in the video-call condition compared to face-to-face. Concurrently, contrary to a mere video, video calls still provide ‘real-time’ communication and we predicted mimicry to be higher in video calls compared to pre-recorded videos. As trust has been previously associated with mimicry [19,77] and has been shown to break down in an electronic context [12], our second hypothesis was that trust would follow the same pattern as that of mimicry. We predicted trust would increase in the face-to-face condition compared to video calls, but it would still be higher in video calls compared to the pre-recorded video. It is assumed that scratching and yawning are emotionally meaningful and convey specific messages [32,78]. Hence, our third hypothesis was that control behaviours would not be mimicked to the same extent as scratching and yawning. Specifically, we predicted mimicry to be higher for the two target behaviours compared to the two control behaviours. Based solely on research that considers mimicry as an affiliative social glue, we would expect mimicry to have positive effects on trust [26,65,66]. However, previous research has shown that mimicry of negative expressions was associated with lower trust [19,67,69], suggesting that mimicry may have a context and behaviour-dependent effect. As the mimicry of scratching and yawning was noted to be greater during tense situations among individuals who are not socially close [55,70], and as our participants were playing a cooperation dilemma with a stranger, our fourth hypothesis was that mimicry of yawning and scratching would have a negative effect on trust.

## 2. Material and methods

### (a) Data statement

Data, materials and code are publicly available on the Open Science Framework at this link https://osf.io/kpb2u/. We report all measures in the study, all manipulations and any data exclusion, and the sample size determination rule.

### (b) Participants

Twenty-seven healthy adults (19 females) between 18 and 34 years old (males: mean ± s.d. = 20.88 ± 1.85; females: 22.26 ± 4.96) voluntarily took part in this experiment at Leiden University (electronic supplementary material, table S7). We aimed to recruit 30 participants within the running time of the experiment (three months during an MSc thesis project). While this is generally considered a small sample size, this was justified by the length of the experiment and technical feasibility. Participants were recruited via SONA Systems. As a consequence of one person dropping out of the experiment halfway, people who correctly guessed the research question (*n* = 2), and people whose task was not completed owing to technical problems (*n* = 4), our final sample included 20 participants (15 females; mean ± s.d. = 22.09 ± 4.61). All participants had normal or corrected to normal vision and hearing and were naive concerning the experiment's hypotheses. None of the participants knew the confederates. All participants gave their written informed consent before the start of the experiment. Participants were compensated with credits and could receive a bonus based on the investment game.

### (c) Design

This study has a repeated-measures within-subject design in which each participant was assigned to all of the following three conditions of the independent variable, namely, interaction context: (i) pre-recorded video, (ii) video call, and (iii) face-to-face. The order of conditions was counterbalanced between participants. All conditions consisted of four trials in which the confederate performed two target behaviours, scratching and yawning [32,78], and two neutral controls, face-touching and lip-biting. Once per trial, the confederate would subtly present each of these behaviours at the same time within the total trial duration (3:40 min). The *confederates* (*n* = 4) were gender-matched with the participants. To ensure timing accuracy, the confederates were instructed via headphones. Within participants, the order of performing these behaviours was the same among the different conditions, although it was fully randomized between participants. In between each trial, a 1 min nature sound was played to both the participants and the confederates. The dependent variables were mimicry, as measured by the occurrence frequency of the aforementioned four behaviours, and trust, as measured by the money participants invested in the trustee (the confederate) after each trial. This investment was taken as an indication of trust.

### (d) Set-up and materials

#### (i) Video materials

The pre-recorded videos consisted of the recordings of the live video call of the previous participant. Thus, the material presented in conditions (i) and (ii) (video call and pre-recorded video) was identical, albeit not among participants. For the first participant, the source of these pre-recorded videos came from a 10 min recording of the confederate from a pilot test. The confederates yawned as naturally as possible for the yawning behaviour, with yawning defined as opening the mouth thoroughly, inhaling air, lifting the shoulders and closing the eyes. Confederates were also instructed to subtly cover their mouth, which is generally considered an act of good manners. Importantly, research has demonstrated that occluding the mouth while yawning does not prevent yawn contagion [29]. For the scratching behaviour, the confederates were asked to bring one hand to the face and rub the skin with the nails. The behavioural instructions were similar for face-touching as for scratching, but the confederates had to touch the face briefly without using the nails. For the lip bite behaviour, the confederates were instructed to slightly bite the lower lip.

#### (ii) Trust game

Participants were instructed to play an investment game with the confederate without referring to the concept of trust. The participant was asked to decide how much of their €10 endowment to issue the other participant (confederate), who was seated at the other squared table, and how much to keep. This investment was then multiplied by a pre-determined amount (×3). Finally, the participant was told that the other participant (confederate) had to decide how much of the increased endowment to return to the participant. The trust game was played after every trial, for a total of 12 trust games. Additionally, the participant also played the trust game before the start of the experiment. The amount invested by the participant in the first trust game served as a baseline level of trust. After that, the trust games were played after each trial to measure the trust level. Crucially, also the confederates were moving at their investment table, but they were not playing for real. In other words, the participant was the only one investing during the investment game.

#### (iii) Setting

**Figure 1.**
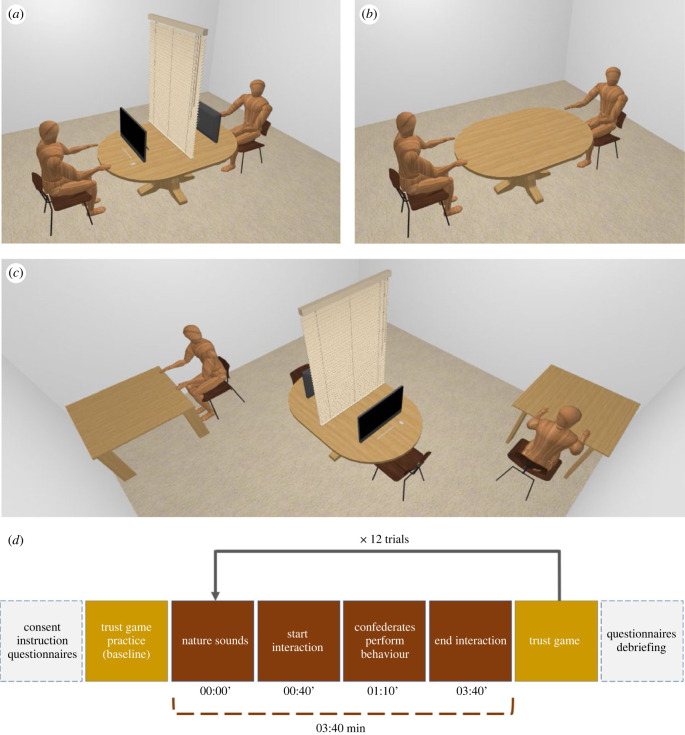
Procedure and setting of the experiment. (*a*) Setting during vide-call and pre-recorded video condition; (*b*) setting during face-to-face conditions; (*c*) setting during the trust game; and (*d*) schematic representation of the experiment task and overview of the study procedure. (Online version in colour.)

The participant and confederate sat opposite each other on a 120 cm table with a blind in the middle and were instructed to count each other's eyeblinks to ensure that the participant attended to the confederate's face. During the pre-recorded condition, two 15.6 inch coloured display laptops were placed 50 cm in front of both persons. Each participant looked at four pre-recorded videos, one per trial. In this condition, the blind was closed to prevent the participant from noticing that the videos were pre-recorded. The same setting with the blind down was arranged during the video call condition, in which the confederate displayed all four behaviours through a video call via the Windows 10 Camera App on the same laptop. Finally, in the face-to-face condition, the blind was open, and the table with laptops was lowered so that the participant and the confederate could look into each other's eyes. On opposite sides of the same room, two squared tables were used to play the investment game: after each trial in each condition, both the participant and the confederate were asked to move to those tables, perform a paper-and-pen investment game and then come back to the interaction table (figure 1). Throughout the three conditions and the investment game, two Canon XA20/25 professional camcorders were used to record potential emotional contagion by the participants. Furthermore, the webcams on both laptops were always on to ensure participants' videos from a frontal perspective.

### (e) Procedure

After reading the information sheet and completing the consent form, the State-Trait Anxiety Inventory (STAI), the Liebowitz Social Anxiety Scale, and Interpersonal Reactivity Index questionnaires were administered (electronic supplementary material), and participants performed a practice trust game. Next, the participant and the confederate were asked to sit at the interaction table, where one of the aforementioned three conditions was performed. They both were asked to count the number of each other's eye blinks for the entire duration of the trial. We thought that this task would reduce the distress associated with looking each other directly in the eye for a prolonged time while also distracting them from the real purpose of the study. Before the start of each trial, both the participant and the confederate put on headphones, through which instructions were given, and closed their eyes for 40 s until an auditory signal was heard. After both opened their eyes, the trial started and the confederates were instructed to display one of the aforementioned behaviours (lip-biting, scratching, yawning, face-touching). To ensure temporal precision in performing the behaviour, this instruction was played 30 s after trial onset (e.g. ‘now, yawn’). After each trial, they wrote down how many eyes-blinks they counted and were asked to return to the squared tables to play the trust game. The procedure was then repeated for the subsequent trials. Between each trial, after the investment game, nature sounds were played. This procedure was kept constant across conditions. See figure 1 for a visualization of the procedure.

At the end of the laboratory session, the participants again completed the STAI questionnaire to measure anxiety levels after the experiment. They also filled in the Percieved Awareness of the Research Hypothesis questionnaire to assess whether participants noticed differences among conditions and in the confederate. The debriefing explained that counting the eye blinks was merely a distraction and that one of the conditions was pre-recorded.

## 3. Statistical analysis

### (a) Mimicry

Two raters coded behaviours using the Behavioural Observation Research Interactive Software [79]. Inter-rater reliability (Cohen's kappa) was 0.95. During the coding, we noticed that the confederates performed more behaviours than instructed. Specifically, they unconsciously showed additional yawns, lip bites, scratches or face touches (electronic supplementary material). Given the presence of these spontaneous behaviours, we decided to adapt the computation of mimicry accordingly. Furthermore, as previous research showed that emotional mimicry fosters synchronicity in dyads [80,81], we also checked whether the confederate unconsciously mimicked the participant back (electronic supplementary material). Considering the length of the trial (180 s), it is possible for mimicry to occur multiple times during a trial. Hence, mimicry is not considered binomial (0–1) but is a cumulative variable that allows for more than one mimicry instance (minimum: 0; maximum observed in the current study: 2). Based on previous studies, we selected a time window of 30 s to count mimicry, being 30 s on average between the time window usually reported for scratch (approx. 5–10 s) and yawn (approx. 60–90) contagion [56,82]. Since this 30 s boundary is admittedly slightly arbitrary and since there is also no complete agreement in the literature [56,83–85], we verified that our results hold when using a different time boundary (electronic supplementary material). Importantly, the results were similar for the different time windows.

All data were analysed using R statistical programming v. 4.0.3 [86]. The dependent variables, mimicry and trust were investigated using a generalized linear mixed model. We fitted both models by inserting one factor at a time and, via log-likelihood tests, determined whether adding a factor significantly improved the model fit. If not, the factor was dropped. For a list of the effect of the non-significant factors, see the electronic supplementary material, table S8.

The first generalized linear mixed model (model 1) was fitted to test the amount of mimicry and the effect of condition and behaviour on mimicry. The multilevel structure was defined by condition (level 1) nested in participants (level 2). We fitted the model using behaviours (yawning, scratching, lip-biting, face-touching) and conditions (face-to-face, video call, pre-recorded video) as fixed factors. As mimicry was following a Poisson distribution, we opted for the R function *glmmTMB* that fitted linear and generalized linear mixed models with various extensions, including zero inflation. *p*-values were corrected for multiple comparisons through the Holm-Bonferroni Sequential correction. To investigate the interaction effect between behaviour and condition on mimicry, we fitted an additional model (model 3) with the same multilevel structure as model 1.

### (b) Trust game

The second linear model (model 2) was fitted to test the possible effect of mimicry, condition and behaviour on trust. First, we computed the dependent variable of trust by subtracting the trust baseline asked before the experiment from the trust investment decision given after each trial. Next, we fitted the model with the *lmer* function using mimicry, behaviour (yawning, scratching, lip-biting, face-touching), and condition (face-to-face, video call, pre-recorded video) as fixed factors, and individual as a random factor. Then, we proceeded with a Tukey post hoc test for each significant effect to estimate contrasts. Finally, we performed the residuals diagnostic. We fitted another model to investigate whether the mimicry of particular behaviours or interaction types affected trust: see model 4 in the electronic supplementary material.

## 4. Results

### (a) Mimicry

**Figure 2.**
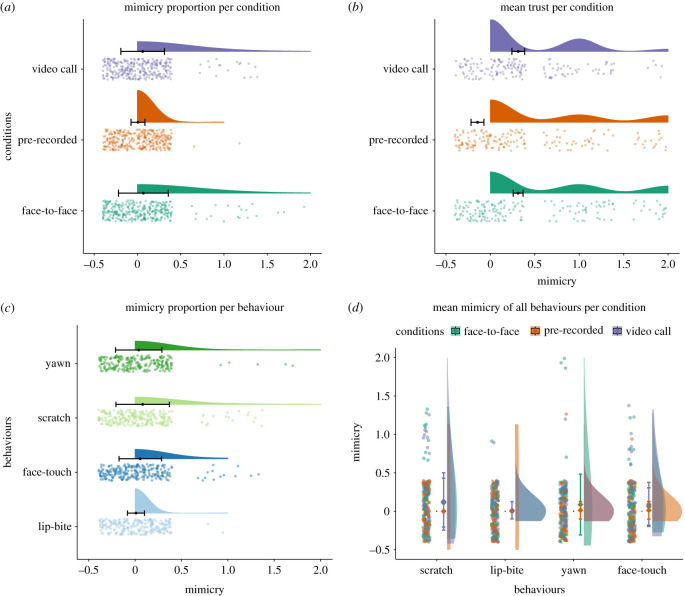
(*a*) Mimicry proportion per condition. Mimicry proportion differed significantly between video call and pre-recorded (*p* = 0.012) and between face-to-face and pre-recorded (*p* = 0.009). (*b*) Mean trust baseline corrected for each condition. Mean trust differed significantly between video calls and pre-recorded (*p* = 0.006) and between face-to-face and pre-recorded (*p* = 0.005). (*c*) Mimicry proportion per behaviours. We observed a significant difference between scratch and lip-bite (*p* = 0.014). (*d*) Interaction effect of behaviour and condition on mimicry. No significant interaction effect has been found. The points represent individual observations, and the error bars represent the mean's standard error (s.e.). (Online version in colour.)

As predicted, we found a significant difference in mimicry between the face-to-face and the pre-recorded video interactions (*β* = −2.3291, s.e. = 0.7970, *p* = 0.009), and between the video-call and pre-recorded video interaction (*β* = −2.2040, s.e. = 0.8021, *p* = 0.012). That is, people mimicked significantly more during a face-to-face interaction and a video call compared to a pre-recorded video. In contrast to our hypothesis, there was no significant difference in the amount of mimicry between the face-to-face and the video-call condition (*β* = −01251, s.e. = 0.4357, *p* = 0.774) (figure 2). Concerning behaviour, partially in line with our hypothesis, the model shows that participants mimicked the target behaviours most. They mimicked scratching more than lip-biting (*β* = 2.2513, s.e. = 0.7434, *p* = 0.014), but no difference was found for yawning compared to lip-biting (*β* = 1.504, s.e. = 0.7817, *p* = 0.217). In contrast to our expectation, there was no difference between scratching and yawning compared to the control behaviour face touch (*p* ≥ 0.05). We did not observe a difference in mimicry rate between the control behaviours (*β* = 1.8718, s.e. = 0.7596, *p* = 0.068), and no differences were found between the two target behaviours (*β* = 0.7472, s.e. = 0.4047, *p* = 0.217) (figure 2). Neither trait anxiety nor empathy influenced the amount of mimicry displayed by the participants. Finally, we investigated the potential interaction between conditions and behaviours (model 3). No significant interaction effect was found (electronic supplementary material). A further cross-validation control analysis supported our effects (electronic supplementary material, analysis).

### (b) Trust

In line with our expectations, we observed a significant negative effect of the pre-recorded video condition on trust (*β* = −0.4114, s.e. = 0.131, *p* = 0.001). A Tukey *post hoc* contrast estimate revealed that participants trusted the confederate significantly more during the face-to-face and the video call compared to the pre-recorded video ((*β* = 0.4114, s.e. = 0.132, *p* = 0.005); (*β* = 0.4040, s.e. = 0.133, *p* = 0.006)). However, in contrast to our hypothesis, the data showed no significant difference in the amount of trust face-to-face compared to video calls (*p* = 0.998). By contrast with our hypothesis, we did not observe a negative effect of mimicry—target and control behaviours combined—on trust (*β* = −0.4570, s.e. = 0.239, *p* = 0.056; electronic supplementary material, table S9). Furthermore, no significant interaction effect was found between behaviours and mimicry (electronic supplementary material, analysis).

## 5. Discussion

Humans rely on a wide variety of nonconscious signals and cues that are inadvertently sent back and forth during an interaction [65]: part of this flow is referred to as mimicry, which contributes to shaping our social perception of others, such as trusting someone or not [87]. As technology has radically changed the way we communicate, it is crucial to understand the impact these changes have on the quality of our interactions. The current study constitutes, to our knowledge, the first experiment to explicitly test the effect of different interaction settings (face-to-face, video calls, pre-recorded videos) on the mimicry of phylogenetically old behaviours and the perceived trustworthiness of a partner. As predicted, the finding shows a significant reduction of mimicry during the pre-recorded video compared to video calls and face-to-face. These findings align with results which show that solely watching a pre-recorded video prevents the nonconscious moment-to-moment tuning of emotional signals that characterize interactions [88]. Conceivably, mimicry of the behaviours observed in the pre-recorded videos is weaker than the other two conditions because it lacks one of the key ingredients: interaction itself (i.e. feedback). While a mere pre-recorded video lacks the synergy of interaction, video calls seem to allow subtle nonverbal reciprocation [1,76]. Although we predicted mimicry to be lower during video calls compared to face-to-face interactions, we did not find a difference between these two conditions. These results differ from published research on the general quality of video calls [89,90], but they are consistent with a multitude of studies finding similar results between face-to-face and video call interactions [91,92]. For instance, Hietanen *et al.* [88] showed that physiological arousal in response to direct gaze is comparable between a video call and face-to-face interactions but significantly lower while watching a pre-recorded video. Emotional mimicry, especially in the case of scratching and yawning, have been strongly associated with changes in arousal in human and other species [47,54]. Within this framework, our findings suggest that the perception of a pre-recorded person on a computer screen might not have the same effect on mimicry—and on accompanying arousal activity—if that perception is not supplied by the mutual exchange of signals and cues (i.e. with a live person on a computer screen and in face-to-face interaction).

Since trust has been strongly associated with mimicry [11,67,87], and trust has been shown to break in electronic contexts [12], we hypothesized trust to show similar patterns as mimicry during these different interactions. We expected trust to be highest in face-to-face interactions, followed by video calls, and lowest in pre-recorded videos. In line with our prediction, we found a significant difference between the pre-recorded video and the other two conditions, but no such difference was observed between video calls and face-to-face interaction. In her work, Rocco [12] has noted that trust succeeded only during face-to-face and breaks in electronic contexts unless a short face-to-face meeting precedes it. Similarly, Verberne *et al.* [73] found that, in the context of a trust game, a mimicking virtual agent was not liked and trusted more than a non-mimicking one [73]. Although our results do not appear to corroborate previous observations, and there was no effect of condition order (electronic supplementary material, tables S3 and S4), they are supported by recent research that found no difference between face-to-face and video call conditions in terms of trust [93]. Taken together, these results seem to suggest that video calls might ensure enough back-and-forth of interaction cues for mimicry to occur, at least in our student population during an interaction between strangers. However, despite the results appearing robust across the tested participants, the current sample size is not sufficient to claim the absence of a difference between video calls and face-to-face interaction in terms of trust. Further investigations are necessary to validate the kinds of conclusions that can be drawn from this study. Nonetheless, it is encouraging to see that trust did not decrease in video calling contexts in the present study, since their use in the workplace, where trust plays a key role, has increased disproportionately in the last years.

Our primary interest was to investigate how the mimicry of some phylogenetically old behaviours that we share with several species could change in the context of new communication technologies. We tested mimicry with scratching and yawning, two behaviours communicating stress and changes in arousal [32,47] that are extremely difficult to suppress [28,29]. Our hypotheses regarding these behaviours were partially confirmed. Both yawning and scratching were significantly more mimicked compared to lip-biting but not compared to face-touching. We expected mimicry to be higher for yawning and scratching, two meaningful behaviours, compared to *both* control behaviours. The present outcome is justified by Chartrand & Bargh [94], who showed that people were more likely to touch their faces when they interacted with a confederate that was touching their faces in turn. Since we touch our faces with high frequency [95,96], spontaneous face-touching is a habit that is extremely hard to control [97]. In fact, the lack of a significant difference between target behaviours and face-touching might be attributed to the high contagiousness of this behaviour. Future research should expand the range of behaviours considered highly contagious. Taken together, our findings do nevertheless suggest that yawning and scratching may have an embedded meaning that is worth spreading among individuals, even via video calls. Moreover, the decrease of mimicry in the pre-recorded condition implies that yawn and scratch contagion might rely on some cues that are lost without live interaction. Yet, video calls seem to provide enough interactional cues for these behaviours to be mimicked.

Regarding our fourth hypothesis, our experiment did not provide conclusive evidence on the relationship between mimicry and trust. Previous literature has shown a negative effect of pupil constriction mimicry on trust [19,69]. However, other studies failed to find an effect of mimicry on trust [71,73,98]. A possible explanation for the present findings might depend on the relatively small sample size. Future research should replicate the study with a larger sample size, ideally calculated through a power analysis. Another possible explanation of the present results lies in the type of behaviours that have been chosen. While scratching has been widely established as a measure of stress [47,59,61], the affiliative outcomes of face-touching mimicry have been replicated multiple times in the literature [65,99]. It is possible that mixing the mimicry of behaviours with different valence might have contributed to the present results. Future studies might consider testing different behaviours in separate experiments to disentangle their effect on trust, perhaps by employing a between-subject design to avoid unwanted interaction effects between behaviours. Concerns with respect to our experimental design can also be raised about yawning. Participants and confederates were instructed to look into each other eyes for a block of 3 min, which is a considerable amount of time to look someone in the eye. In fact, some participants reported a conscious suppression of the urge to express the behaviours, yawning in particular. The majority of them reported that they felt uncomfortable with blatantly yawning while being directly observed. This claim is supported by previous research showing that people inhibit yawns more easily if aware of being observed [28]. Regardless of the limitations, it is worth noticing that neither our results corroborate the negative effect of mimicry on trust [19,69], *nor* did the previous studies find a positive effect [19,66]. Owing to the substantial fragmentation of the literature on the topic, framing the function of mimicry as solely affiliative might be reductive. For instance, a recent study showed that some types of yawning might be linked with more affiliative intents, while others with more agonistic and tense situations [35]. Similarly, mimicry of pupil dilation can lead to more trust, but the effect was in the opposite direction for pupil constriction mimicry [69]. Clearly, more research is needed to disentangle the effect of the mimicry of different behaviours on social decisions. We call for more experimental designs looking at the effect of different behaviours on mimicry, but also at the different social contexts. This will be beneficial to shed light on whether it is mimicry itself that enhances our trust in others or rather the embedded meaning of the mimicked behaviours.

## 6. Limitations

The results of the present study should be interpreted with caution, as the study presents some limitations. First, participants and the confederate were always in the same laboratory in every condition. Since sharing the environment is unusual in the context of a video call, this could have contaminated our results. Scratching and yawning have been shown to increase the level of vigilance or arousal within the group to eventually prepare for environmental changes [38,54], and the presence of the partner may be a prerequisite for the mimicry of these behaviours to occur. While a study on *Macaca thibetana* (Tibetan macaques) showed that scratching contagion is higher in individuals that are spatially close [88], yawning research suggests that spatial proximity may not be the most important factor mediating mimicry (gelada baboons [43], wolves [45]). The research on spatial proximity in yawning and scratching has primarily focused on non-human animals, and further research is needed to investigate how much sharing environment affects mimicry of those behaviours in human interactions. There is substantial ground in the literature to believe that mimicry would also occur without direct spatial proximity. Lakin & Chartrand [65] found that, although meditated by a conscious affiliation goal, mimicry of face-touching was still occurring during video calls [61]. Numerous studies also detected the presence of mimicry with virtual agents where the spatial proximity is not in place [68,73,89]. These results suggest that mimicry may arise when the individuals are spatially close. Future research should consider adding a critical condition in which participants are tested in spatially separated settings, perhaps comparing it to when they are in the same room. Nonetheless, the fact that mimicry is happening via a screen—which is a recent way of communicating in evolutionary terms—is still valuable information. A further methodological issue is that the confederates performed more behaviours than they should have. As such, the traditional method of counting mimicry, using pre-defined epochs mainly implemented with computer tasks, did not fit our requirements. That is, it does not consider the presence of another human being that mimics the participants' back. Future studies should consider more ecologically friendly settings that do not denaturalize the back-and-forth flow of behaviours, which ultimately is the heart of human social interaction. Furthermore, the present study is also limited by its relatively small sample size and recruitment procedure. Although the cross-validation analysis indicated our results to be robust, the sample size might be too small to draw a firm conclusion about the relationship between trust and mimicry in the present study. Moreover, we recruited participants from the Leiden University recruitment platform (SONA System). This platform allows recruiting of mostly university students, which is a rather homogeneous sample in terms of demographic (i.e. age, education, cultural background) and experience with technology (i.e. video calls). Future research should consider a more varied sample, including participants of different ages, education levels, and cultural backgrounds, perhaps controlling for previous experience with technology.

## 7. Conclusion

In the present study, we examined how much video calls compared to face-to-face interactions and pre-recorded videos impact the mimicry of multiple behaviours and trust in a partner. Although participants shared the same environment in every condition, we showed that meaningful behaviours like scratching and yawning are mimicked through a screen during video calls, roughly to the same extent as face-to-face interaction. Similar to mimicry, trust was not different during face-to-face interactions and video calls. However, mimicry did not have a role in shaping trust in others. The evidence of this study points towards the idea that video calls may be underrated as a means of communication, as they might still provide access to a large number of non-verbal signals that shape our social perception of others. In our view, these results constitute a promising initial step towards a deeper understanding of how malleable our social interaction is.

## Data Availability

The data are provided in the electronic supplementary material [100].
